# Dysbiotic Gut Microbiota and Dysregulation of Cytokine Profile in Children and Teens With Autism Spectrum Disorder

**DOI:** 10.3389/fnins.2021.635925

**Published:** 2021-02-10

**Authors:** Xia Cao, Kevin Liu, Jun Liu, Yen-Wenn Liu, Li Xu, Hua Wang, Yunhui Zhu, Pengfei Wang, Zhiwei Li, Jie Wen, Chen Shen, Meng Li, Zuqing Nie, Xue-Jun Kong

**Affiliations:** ^1^Second Affiliated Hospital of Kunming Medical University, Kunming, China; ^2^Athinoula A. Martinos Center for Biomedical Imaging, Massachusetts General Hospital, Charlestown, MA, United States; ^3^Institute of Biochemistry and Molecular Biology, National Yang-Ming University, Taipei, Taiwan; ^4^Hong-Ta District Maternal and Child Health Hospital, Yuxi, China; ^5^Department of Psychiatry, Beth Israel Deaconess Medical Center, Boston, MA, United States

**Keywords:** autism spectrum disorder, gut microbiota, dysbiosis, inflammation, cytokine, short chain fatty acid, butyrate

## Abstract

Inflammation and the gut-brain axis have been implicated in the pathogenesis of autism spectrum disorders (ASDs). To further understand the relationship between aberrant immune responses and dysbiotic features of the gut microbiome in ASD, we enrolled 45 ASD individuals and 41 healthy control subjects with ages ranging from 2 to 19 years. We found that ASD group subjects have significantly higher plasma levels of IL-2, IL-4, IL-5, IL-6, IL-10, TNF-α, TNF-β, and IFN-γ when compared to healthy controls (FDR-adjusted *p* < 0.05). The plasma levels of pro-inflammatory cytokines IFN-γ and IL-6 are found to be further associated with several largely pathogenic gut microbiota uniquely detected in subjects with ASD. Furthermore, the ASD gut microbiome is characterized by reduced levels of several beneficial microbiota, including *Bacteroides* (FDR-adjusted *p* < 0.01) and Lachnospiraceae (FDR-adjusted *p* < 0.001). Analysis of Lachnospiraceae family and genus level taxa suggested that relative abundances of such taxa are negatively correlated with pro-inflammatory signaling cytokines IFN-γ and IL-6, particularly in subjects with severe ASD as defined by CARS (*p* < 0.05). Several largely pathogenic genera are determined to be associated with the pro-inflammatory cytokines IFN-γ and IL-6 (FDR-adjusted *p* < 0.1). Additionally, IL-4 is significantly negatively correlated with CARS total score (*p* < 0.05). Based on such results, we propose that the association between the disturbances of specific cytokines and alterations in gut microbiota abundance observed in children and adolescents with ASD provides additional evidence on the induction of aberrant pro-inflammatory mechanisms in ASD and its early diagnosis.

## Introduction

Autism spectrum disorders (ASDs) is a behaviorally defined neurodevelopmental disorder that has demonstrated a rapid increase in prevalence over recent years (Baio et al., 2018). While the pathogenesis of ASD remains largely unclear, altered gut microbiome compositions were found to be associated with ASD and has gained a significant amount of attention in the field. Gastrointestinal (GI) symptoms and gut microbiome dysbiosis are common comorbidities seen in individuals with ASD and are known to be correlated with the behavioral severity of ASD (Wasilewska and Klukowski, 2015; Fattorusso et al., 2019). In particular, dysbiosis of the gut microbiome is associated with the pathogenesis of inflammatory conditions such as inflammatory bowel disease (IBD) (Rosenfeld, 2015; Niu et al., 2019). As attempts to characterize the disturbed ASD microbiome, previous studies have indicated increased amounts of pathogenic bacteria, such as select species from the *clostridium* genus, and reduced amounts of beneficial bacteria, such as *Bifidobacterium* (Rosenfeld, 2015; Niu et al., 2019). Some of these signature changes showed potential utility in assisting early diagnosis of ASD and in the characterization of ASD subtypes (Kong et al., 2019). Moreover, probiotics treatment was found to improve ASD behaviors in both animal models and clinical studies, which serves as a potential therapeutic option for alleviating GI symptoms and reducing aberrant behaviors associated with ASD (Hsiao et al., 2013; Sgritta et al., 2018; Sanctuary et al., 2019). As early diagnosis of ASD is known to improve long-term prognosis of affected individuals and difficulties for clinicians to identify socio-behavioral abnormalities in children younger than 12 months of age, there is a rising need for biological diagnostic markers of ASD (Mandell et al., 2005; Pierce et al., 2009). As a result, studies on the elucidation of biological diagnostic biomarkers of ASD in susceptible infants are conducted in some countries as young as 6–12 months (Emerson et al., 2017; Jones et al., 2017).

Previous studies have suggested that inflammatory mechanisms were implicated in the pathogenesis of ASD, at least within a subset of patients, where inflammation-induced microglial cell activation in central nervous system (CNS) infections has been proposed to result in the underconnectivity of the ASD brain (Rodriguez and Kern, 2011). Such hypotheses are consistent with findings of aberrant immune functions in individuals with ASD, as pro-inflammatory cytokines were found to be elevated in both blood and cerebrospinal fluid (CSF) of ASD individuals (Goines and de Water, 2010). While the cause of inflammation and elevated pro-inflammatory cytokines in ASD remains unclear, several studies have reported that elevated levels of intracellular cytokines and pro-inflammatory cytokine receptors in peripheral blood mononuclear cells (PBMCs) in ASD is associated with dysregulation of immune cell signaling and expression of associated surface cytokine receptors, thus providing additional insight into the molecular perspective of ASD immune abnormalities (Ahmad et al., 2018, 2019a, b, 2020; Ansari et al., 2020; Nadeem et al., 2020a, b). Additionally, previous studies on the ASD gut microbiome have suggested that dysbiosis in the gut microbiome may contribute to the aberrant brain development processes (Li et al., 2017). Thus, regulation of the gut microbiome may provide a twofold benefit to the alleviation of both ASD core symptom severity and associated GI problems commonly found in ASD through the reduction of inflammation. Furthermore, microbial metabolites such as neurotransmitters and short-chain fatty acids (SCFAs) directly interact with the gut-brain signaling axis and mediate immune responses. Short-chain fatty acids are essential to intestinal health, with butyrate being the major energy source for colonic cells. Specifically, butyrate has been found to possess anti-inflammatory properties, enhance epithelial barrier integrity, and protect against enteric pathogen colonization and infection by multiple mechanisms (Liu et al., 2018). Previous studies on the utility of using butyric acid-producing bacteria as probiotics have attracted attention and have led to the exploration of the use of such bacteria as therapeutic agents for IBD (Immerseel et al., 2010; Wang et al., 2013). Butyrate was also reported to protect the brain and enhance neuroplasticity in neurological disease models (Val-Laillet et al., 2018). There has been a reported decrease in the abundance of key butyrate-producing bacteria, which may contribute to the pro-inflammatory processes in ASD resulting from altered SCFA and fatty acid metabolism (Liu et al., 2019). Furthermore, studies on prebiotics have suggested that supplementation with galactooligosaccharide (GOS) may promote the flourishing of *Bifidobacterium* and increase *in vitro* SCFA production (Grimaldi et al., 2016).

In the present study, we leverage the combination of stool microbiome 16s rRNA sequencing and plasma multiplex cytokine immunofluorescence profiling to examine the interaction between gut microbiome dysbiosis and systemic inflammation in individuals with ASD. First, we compared the peripheral blood plasma cytokine levels between individuals with ASD and healthy controls to identify groupwise differences in cytokine expression. Subsequently, we compared the gut microbiota between individuals with ASD and healthy controls to identify the differential abundances of pathogenic and beneficial taxa between the two groups, with particular attention paid to microbial taxa implicated in the inflammatory pathways. Through such comparisons, we attempt to assess the clinical significance of found dysbiotic species within the gut microbiome of individuals with ASD and their associations with blood plasma cytokine concentrations. Such comparisons enable the further understanding of systemic inflammation, gut and brain inflammation, and interference on the gut-brain signaling axis. To our knowledge, the modulatory effects of systemic inflammation associated with butyrate-producing microbiota within the gut microbiome have not been previously explored in individuals with ASD. Subsequently, we investigated the correlation between cytokines, pathogenic microbiota present in the gut of ASD individuals, and clinical indices of ASD core symptoms, which serves as an initial effort to uncover potential microbiome-cytokine biomarkers for clinical subtyping in individuals with ASD.

## Materials and Methods

### Ethics and Consent

This study was approved by the Institutional Review Board (IRB) of the second affiliated hospital of Kunming Medical University (Review-YJ-2016-06) and was conducted in accordance with the Declaration of Helsinki. Written informed consent was obtained from the parents or legal guardians of study participants based on the IRB protocol requirements prior to participation in the study. A copy of the written consent is available for review upon request.

### Participants and Autism Diagnostic Observation

The study enrolled 45 drug-naïve subjects with a clinical diagnosis of ASD who were recruited from the Psychiatric Department of the Second Affiliated Hospital of Kunming Medical University and 41 healthy controls were recruited from Yuxi city, China. ASD group subjects were screened based on inclusion and exclusion criteria. Subjects who scored 6 or greater based on the Autism Mental Status Exam (AMSE) and those who scored 30 or greater based on the Childhood Autism Rating Scale (CARS) were then administered a comprehensive evaluation by a child and adolescent psychiatrist. The best estimate clinical diagnosis (BECD) protocol was then applied to indicate whether the required ASD characteristics of DSM-5 diagnostic criteria were met; in such cases, the psychiatrist then collects medical history (chief complaint, history of present diseases, past medical history, auxiliary examination results, and developmental milestones) upon review of the two respective assessment tool results and having interviewed the caregivers and patients. Participants who scored between 30 and 36 on the CARS were classified as mild to moderate autism and those with scores between 37 and 60 points were classified as severe autism. Healthy controls were similarly screened by a psychiatrist and through ASD psychometric tests to exclude participants that fulfill the diagnostic criteria of ASD, attention deficit/hyperactivity disorder (ADHD), oppositional defiant disorder (ODD), psychiatric developmental delay, conduct disorders, and other common neurodevelopmental and psychiatric disorders seen in children and adolescents.

### Inclusion and Exclusion Criteria

#### Inclusion Criteria

1. Pediatric patients aged 2–19 years old;
2. Meets the diagnosis criteria based on autism assessment scales and diagnostic protocols, including The Diagnostic and Statistical Manual of Mental Disorders, Fifth Edition (DSM-5), Childhood Autism Rating Scale (CARS), the Autism Mental Status Exam (AMSE), and Clancy Autism Behavior Scale (CABS).

#### Exclusion Criteria

1. Having concurrent neurological or psychiatric disorders, or cognitive impairment, or those uncooperative;
2. Currently taking any psychiatric medications;
3. Having taken or ingested antibiotics, yogurt, or probiotic products, as well as other medications or foods with immunoregulatory effects;
4. Having concurrent heart, lung, kidney, or other major organ abnormalities;
5. Having concurrent serious systemic diseases that are not well controlled;
6. Currently participating in other clinical trials or having participated in other clinical trials that involve administration of any medications within less than one month before participating;
7. Suspected or confirmed alcohol or drug abuse history, or other conditions that may present complications in the determination of eligibility based on the investigator’s judgment;
8. Patients that are not suitable for participation based on the investigator’s judgment.

### Blood Sample Collection

Peripheral blood was drawn and collected into EDTA containing tubes (3 ml) from all participants after informed consent was obtained. Samples were subsequently centrifuged at 3000 rpm for 10 min for fractionation. The plasma fraction was collected, dispensed into five EP tubes (1.5 ml), and stored at −80°C conditions until further processing.

### Plasma Cytokine Concentrations Measurements by Bead-Based Immunoassays

Plasma levels of IL-1β, IL-2, IL-4, IL-5, IL-6, IL-8, IL-10, IL-17A, IL-17F, TNF-α, TNF-β, IFN-γ, IL-22, and IL-12 p70 were quantified by a Human 14-plex kit (AimPlex, Cat# C191114) and a bead-based array protocol. All experimental processes were conducted according to the manufacturer’s instructions. Antibody-beads were added into the 96 wells, standards and samples were dispensed into the wells, and all plasma samples and standards were incubated on the arrays at room temperature with gentle shaking. Biotinylated antibodies were then added after several washes and the arrays were incubated at room temperature. The streptavidin-PE cocktail was added. After another series of washes and incubations, the reading buffer was added, and results were collected via a flow cytometer. Finally, the cytokine concentration was calculated using the FCAP arrayTM software V3.0 (BD Company).

### Stool Sample Collection

Stool samples were collected using the MGIEasy Fecal DNA samples collection kit (MGI tech, Cat# A0028). Parents of the study participants were provided with the stool collection kit for at-home sample collection. While wearing gloves, parents of the study participants were instructed to place the plastic stool collection sheets under the toilet seat and collected feces were then sampled and transferred to the sample storage tube containing microbial preservation solution using a provided sterile cotton swab. The sample tube lid is then tightened for storage at room temperature until processing and sequencing.

### DNA Extraction and Amplicon Sequencing

#### Extraction of Genomic DNA

Total genomic DNA from samples was extracted using DNeasy PowerSoil Kit (QIAGEN). DNA concentration and quality were checked using a NanoDrop Spectrophotometer. DNA was diluted to 10 ng/μl using sterile ultrapure water and stored at −80°C for downstream use.

#### Polymerase Chain Reaction (PCR)

The V4 region of 16S rRNA genes was amplified using the 515F(5′-GTGYCAGCMGCCGCGGTAA-3′)-806R(5′-GGACTACHVGGGTWTCTAAT-3′) primer pair with 12 nt unique barcodes. The PCR mixture (25 μl) contained 1x PCR buffer, 1.5 mM MgC_*l2*_, each deoxynucleoside triphosphate at 0.4 μM, each primer at 1.0 μM, 0.5 U of KOD-Plus-Neo (TOYOBO) and 10 ng template DNA. The PCR amplification program consists of initial denaturation at 94°C for 1 min, followed by 30 cycles (denaturation at 94°C for 20 s, annealing at 54°C for 30 s, and elongation at 72°C for 30 s), and a final extension at 72°C for 5 min. Three replicates of PCR reactions for each sample were then combined.

#### PCR Product Detection

PCR products mixed with 1/6 volume of 6X loading buffer were loaded on 2% agarose gel for detection. Samples with a bright main band between 200 and 400 bp were chosen for further experiments.

#### PCR Product Purification, Quantification, and Mixing

The electrophoresis band was purified using the OMEGA Gel Extraction Kit (Omega Bio-Tek, United States). DNA was quantified using Qubit 2.0 Fluorometer (Thermo Scientific). PCR products from different samples were pooled with equal molar amounts.

#### Library Preparation, Sequencing, and Data Availability

Sequencing libraries were generated using TruSeq DNA PCR-Free Sample Prep Kit following the manufacturer’s recommendations and index codes were added. The library quality was assessed on the Qubit 2.0 Fluorometer (Thermo Scientific) and Agilent Bioanalyzer 2100 system. At last, the library was applied to paired-end sequencing (2 × 250 bp) with the Illumina Hiseq apparatus at Rhonin Biosciences Co., Ltd. All raw data from 16s rRNA Illumina amplicon sequencing have been deposited in The National Center for Biotechnology Information (NCBI)–Sequence Read Archive (SRA) database with the BioProject accession number PRJNA642975.

### Bioinformatics Processing and Analysis of Amplicon Sequencing Data

Bioinformatics processing of amplicon sequences was performed using the *bioBakery workflows* v0.13.2 16s *VSEARCH* pipeline along with manual *R* scripts (*R* v3.6.1, *RStudio* v1.2.5019) (McIver et al., 2017). Quality filtering was conducted using a Phred score threshold of 20 and sequences that are less than 75% in length relative to the initial length are discarded. Closed reference operational taxonomic unit (OTU) picking was performed using *Vsearch* v2.14.1 with *Greengenes* v13.8 (97% sequence similarity) 16s rRNA database. Genus level OTU alpha diversity of microbial communities was estimated using the Shannon index. Normalization by copy number and predictive functional profiling of gut microbiota metagenome was conducted via *PICRUSt* v1.1.4 as a part of the *bioBakery* 16s workflow (Langille et al., 2013).

Microbiome co-abundance network analysis was performed using the NAMAP with Spearman’s rank-correlation algorithm via MetagenoNets to elucidate ecological interactions between bacterial features (Nagpal et al., 2020). Features were filtered using a prevalence cutoff of ≥0.0015 of maximum prevalence and with occurrences in ≥10% of samples. After feature filtration and normalization, 50 features remained. Taxa abundances were subsequently normalized via Total Sum Scaling (TSS). All plotted network correlations are significant at *p* < 0.05 based on bootstrapping of 100 iterations.

### Statistical Analysis

The Wilcoxon rank-sum test was used to compare means of all continuous outcomes, including subject demographic characteristics and plasma cytokine concentrations, between ASD and control groups. For differences in mean subject demographic characteristics, results were considered significant at a significance threshold of *p* < 0.05. Additionally, false discovery rate-adjusted *p-*values (FDR-adjusted *p-*values, *Q*-values) were computed to account for false positives due to multiple testing in the comparison of mean plasma cytokine concentrations between groups and statistical significance is evaluated using a cutoff of *Q* < 0.1. *MaAsLin2* was used to explore both the intergroup differences in microbial relative abundance and functional composition of the microbial metagenome (Mallick et al., 2020). Furthermore, Spearman’s rank correlation was used in conjunction with *MaAsLin2* in the identification of per-feature correlations between clinical indices, cytokine concentrations, and microbial relative abundances.

Following the identification of gut microbiota associated with gut microbiome dysbiosis, we conducted logistic regression analysis to evaluate the performance of using identified dysbiotic gut microbiota in classifying the severity subgroups of ASD, which is based on the CARS definition of severity categories, including *mild-to-moderate* and *severe*. Based on the fitted logistic regression model, we constructed receiver operated characteristics (ROC) curve and computed the areas under the curves (AUCs) using the empirical method based on the *ROCit* package in *R.* In addition, 95% confidence intervals (CIs) were computed using 200 bootstrapping iterations.

## Results

### Demographics and Characteristics of the Participants

We recruited 45 children and adolescents with ASD and 41 healthy controls. ASD group subjects consisted of 36 male and 9 female individuals aged 6.80 ± 3.79 years, while the healthy control group consisted of 34 male and 7 female individuals aged 5.16 ± 0.99 years. Age and BMI was found to be significantly different between groups and the age and BMI distributions are shown in Supplementary Figure 1 (Wilcoxon rank-sum test, *p* < 0.05). No significant groupwise differences were observed for sex, height, and weight (Pearson’s *χ*^2^-test and Wilcoxon rank-sum test). A summary of the subject demographics and characteristics is presented in Table 1.

**TABLE 1:** Summary of subject demographics and characteristics.

	ASD (*n* = 45)	Control (*n* = 41)	Total participants (*n* = 86)
Age (mean ± SD, years)	6.80 ± 3.79	5.16 ± 0.99	6.02 ± 2.93
**Sex**			
Male (*n*)	36 (80%)	34 (82.93%)	70 (81.4%)
Female (*n*)	9 (20%)	7 (17.07%)	16 (18.6%)
Height (mean ± SD, cm)	117.91 ± 21.29	113.60 ± 8.24	115.86 ± 16.47
Weight (mean ± SD, kg)	24.52 ± 13.29	19.76 ± 4.42	22.25 ± 10.31
Body mass index (BMI, mean ± SD)	16.55 ± 2.43	15.17 ± 1.70	15.89 ± 2.22
**Childhood autism rating scale (CARS)**			
Total Score (mean ± SD)	36.67 ± 4.56	–	–
Mild-to-moderate (*n*)	25	–	–
Severe (*n*)	18	–	–

### Disturbances of Plasma Inflammatory Markers in Children and Teens With ASD

To elucidate the interactions between gut microbiota and inflammatory cytokines in individuals with ASD, we first assayed the plasma concentrations of cytokines for subjects with ASD and healthy controls. Analysis of all assayed plasma cytokines suggested that IL-2, IL-4, IL-5, IL-6, IL-10, TNF-α, TNF-β, and IFN-γ are significantly elevated in ASD group subjects when compared to controls (Table 2, Wilcoxon rank-sum test, FDR-adjusted *p* < 0.05). No significant differences were found between groups for IL-1β, IL-8, IL-12 p70, IL-17A, IL-17F, and IL-22. Furthermore, we performed correlational analysis between each of the 14 assayed plasma cytokines against body mass index (BMI) to assess for potential relationships between inflammation and weight status. IL-1β was found to be positively associated with BMI with statistical significance in all subjects (Supplementary Figure 2A, Spearman’s ρ = 0.29, *p* < 0.05). Interestingly, while such a correlation displays statistical significance using all subjects, the association between plasma IL-1β and BMI is not observed in healthy controls (Spearman’s ρ = 0.069, *p* = 0.78) and shows borderline significance in subjects with ASD (Supplementary Figure 2B, Spearman’s ρ = 0.35, *p* = 0.057).

**TABLE 2:** Summary of peripheral blood plasma cytokine concentrations in children with ASD and healthy controls.

	Plasma concentration (mean ± SD, pg/ml)		*p*-value	*Q*-value^1^
	ASD (*n* = 45)	Control (*n* = 41)		
IL-1β	2.30 ± 0.65	2.15 ± 0.93	0.136	0.212 NS
IL-2	31.23 ± 44.32	3.58 ± 1.88	<0.0001	< 0.0001****
IL-4	8.88 ± 7.04	5.26 ± 4.19	0.00723	0.0145*
IL-5	3.92 ± 2.14	2.71 ± 1.30	0.0179	0.0312*
IL-6	20.54 ± 18.74	5.54 ± 4.06	0.000137	0.000638***
IL-8	12.75 ± 5.32	13.23 ± 4.97	0.562	0.562 NS
IL-10	9.64 ± 5.06	6.04 ± 2.73	0.000309	0.00108**
IL-12 p70	6.39 ± 2.16	5.70 ± 0.71	0.332	0.423 NS
IL-17A	4.75 ± 4.44	4.33 ± 2.12	0.525	0.562 NS
IL-17F	4.72 ± 1.71	4.58 ± 2.08	0.529	0.562 NS
IL-22	1.13 ± 0.64	1.03 ± 0.84	0.263	0.368 NS
TNF-α	6.92 ± 5.65	3.91 ± 1.63	0.00295	0.00689**
TNF-β	4.50 ± 1.46	3.05 ± 1.01	<0.0001	0.000189***
IFN-γ	5.96 ± 3.49	3.50 ± 0.60	0.00112	0.00314**

^1^NS: non-significant at an FDR-adjusted p-value (Q-value) cutoff of 0.05; *Q < 0.05, **Q < 0.01, ***Q < 0.001, ****Q < 0.0001.

### Dysbiosis in the Gut Microbiome of Children With ASD

**FIGURE 1:**
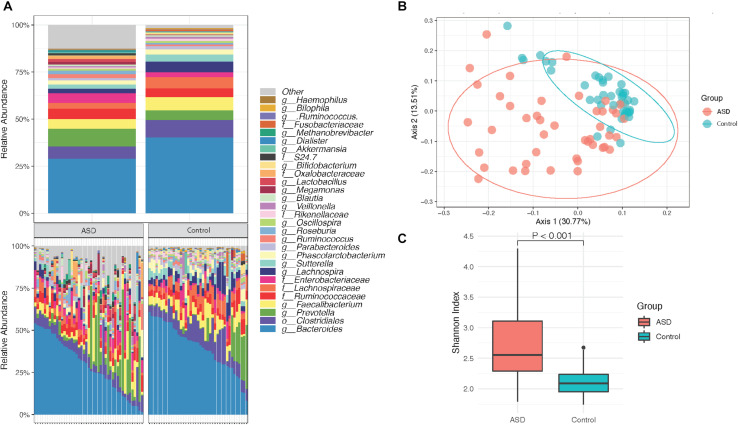
Overview of groupwise subject gut microbiota composition. **(A)** Mean relative abundance of the top 30 taxa in subjects with ASD (*n* = 45) and healthy controls (*n* = 41). **(B)** PCoA of gut microbial β diversity (Bray–Curtis) with 95% confidence ellipses after filtering. **(C)** Alpha diversity via the Shannon index displays significant differences between subjects with ASD (*n* = 45) and healthy controls (*n* = 41). Taxa were agglomerated at the genus level and filtered using a prevalence threshold of ≥0.01% abundance and an occurrence threshold of ≥10% of all samples.

Assessment of the gut microbiome profiles of subjects with ASD and healthy controls were performed following agglomeration of identified OTUs at the genus level and filtering of identified taxa using a prevalence threshold of ≥0.01% abundance and an occurrence threshold of ≥10% of all samples. A total of 3347 taxa were identified and 686 taxa remained after filtering. Based on our findings, the gut microbial composition in subjects with ASD contains several genera that are differentially abundant relative to controls, with control group individuals having a larger relative abundance of bacteria from the top 30 most abundant species (Figure 1A). The difference in gut microbiota composition is evident in the clustering behavior of β diversity based on Bray–Curtis dissimilarity between ASD group and controls (Figure 1B). In contrast to previous studies that have reported reduced α diversity within the ASD gut microbiome as compared to control group individuals, our subject population suggested an increase in α diversity among ASD group individuals (Figure 1C, Wilcoxon rank-sum test, *p* < 0.001).

**FIGURE 2:**
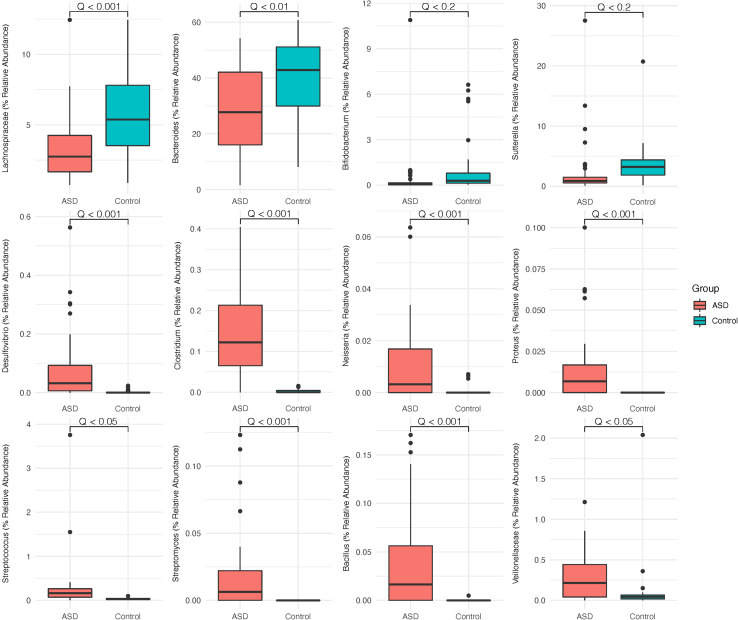
Groupwise differential enrichment of gut bacterial genera. Taxa abundances of 45 subjects with ASD and 41 healthy controls agglomerated at the genus level and filtered using a prevalence threshold of ≥0.01% of the maximum prevalence and occurring in ≥10% of all samples. FDR-adjusted *p*-values (*Q-*values) are labeled above the respective horizontal comparison lines.

To characterize potential clinically significant bacteria that are associated with the microbiome profile of subjects with ASD, we identified several bacterial genera and families that have been previously implicated to possess either pathogenic or beneficial roles in the ASD gut microbiome. Using *MaAsLin2* to explore the intergroup differences of gut microbiota relative abundance, we found that several known beneficial bacterial genera and families are differentially enriched in the control group as compared to the ASD group. The relative abundance of the *Bacteroides* genus (FDR-adjusted *p* < 0.01) and the Lachnospiraceae family (FDR-adjusted p< 0.001) were found to be reduced in the ASD group gut microbiome relative to controls (Figure 2). The *Bifidobacterium* (FDR-adjusted *p* < 0.2) genus is absent from the majority of our study population, with more control group individuals displaying higher levels of such bacteria as compared to that of the ASD group.

Next, we identified bacterial genera that are enriched in the ASD group microbiome, several of which are pathogenic taxa known to dominate the microbiome of subjects with ASD (Figure 2). We found that ASD group subjects possess significantly higher abundances of *Clostridium* (FDR-adjusted *p* < 0.001), *Desulfovibrio* (FDR-adjusted *p* < 0.001), and *Streptococcus* (FDR-adjusted *p* < 0.05). Higher abundance of *Neisseria*, *Bacillus*, and *Streptomyces* are also observed in ASD group subjects, while few to none of the healthy controls had detectable levels of such genera (FDR-adjusted *p* < 0.001).

**FIGURE 3:**
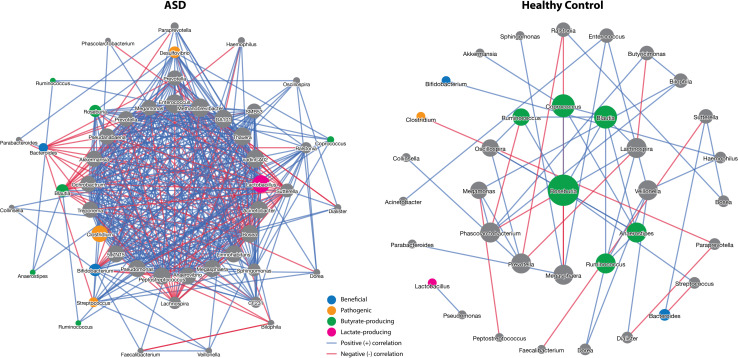
Groupwise genus level NAMAP with Spearman’s rank correlation network of gut microbiota. Gut microbiota abundances of 45 subjects with ASD and 41 healthy controls were agglomerated at the genus level and filtered to include taxa with a prevalence of ≥0.15% of the maximum prevalence and occurring in ≥10% of all samples, such that 50 features remained. Shown associations are statistically significant using a cutoff of *p* < 0.05 using 100 bootstrapping iterations. Colors of nodes indicate identified microbiota characteristics of interest.

In the elucidation of ecological interactions between the identified differentially abundant microbiota, we examined the co-abundance network for microbiota features among both ASD and control group subjects. To limit the number of plotted taxa for ease of visualization, we applied more rigorous taxa filtering criteria. All OTU abundances were agglomerated at the genus level and filtered to include taxa with a prevalence of ≥0.15% of the maximum prevalence and occurring in ≥10% of all samples. After feature filtration and normalization, 50 features remained. Among the 50 remaining features, 49 nodes were observed to be significant in the ASD group network, while 36 nodes were observed in the control group network. Furthermore, 443 edges were observed in the ASD group network as compared to the 54 edges observed in the control group network. All observed correlations are significant at *p* < 0.05 (Figure 3).

**FIGURE 4:**
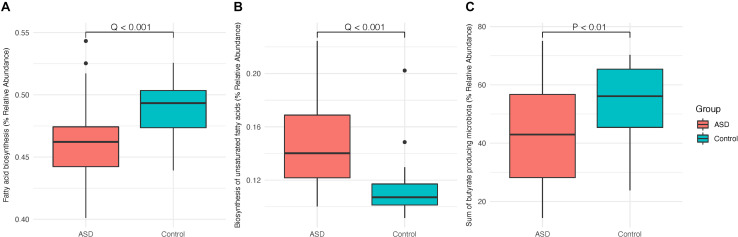
The ASD gut microbiome is characterized by a significant reduction in butyrate-producing microbiota. **(A,B)** Differentially expressed fatty acid-associated metabolic functions predicted based on the 16s profile of the gut microbiota in subjects with ASD (*n* = 45) and healthy controls (*n* = 41). **(C)** Sum of butyrate-producing microbial taxa relative abundances in subjects with ASD (*n* = 45) and healthy controls (*n* = 41). *Q-* and *p*-values are labeled above the respective horizontal comparison lines.

Using predictive functional profiling, we then assessed the relative composition of bacteria that are involved in processes associated with the production of SCFAs, such as the saturated SCFA, butyrate. The biosynthesis of fatty acids overall is found to be significantly less in the ASD group subjects when compared to controls while biosynthesis of unsaturated fatty acids is increased in ASD group subjects (Figures 4A,B, FDR-adjusted *p* < 0.001). By inference, the gut microbiome of individuals with ASD harbor lower levels of saturated fatty acids, such as butyric acids and other SCFAs, compared to healthy controls. Comparing the sum of all considered butyrate-producing genera between ASD and control groups, we found that ASD group subjects display lower levels of butyric acid-producing bacteria overall (Figure 4C, Wilcoxon rank-sum test, *p* < 0.01), which is consistent with assessments via predictive functional profiling. The detected and considered butyrate-producing genera include *Anaerostipes*, *Bacteroides*, *Blautia*, *Coprococcus*, *Eubacterium*, *Roseburia*, and *Ruminococcus*; the families include Lachnospiraceae and Ruminococcaceae.

### Associations Between Plasma Cytokines, Gut Microbiota, and ASD Severity

**FIGURE 5:**
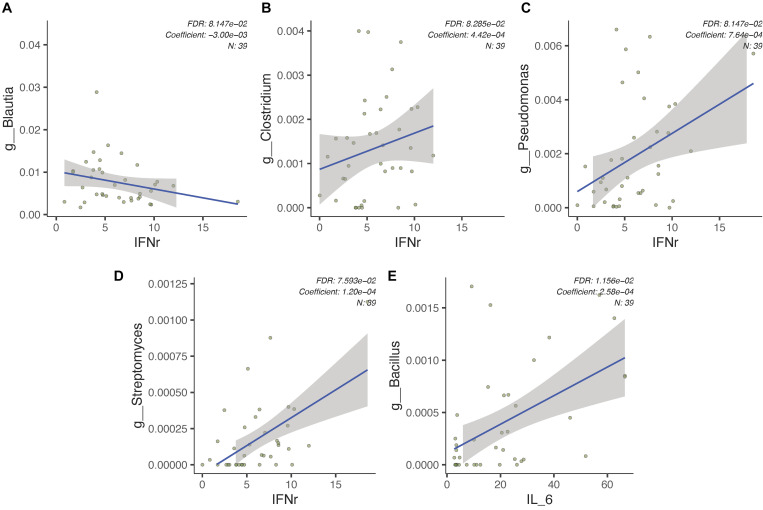
Gut microbiota in subjects with ASD is associated with pro-inflammatory signaling cytokines. **(A–D)** Relative abundances of several bacterial genera found within the gut microbiome of subjects with ASD (*n* = 39) are associated with plasma IFN-γ levels. **(E)** *Bacillus* relative abundance is positively correlated with plasma IL-6 levels in subjects with ASD (*n* = 39).

In an attempt to determine the association between plasma cytokine levels, gut microbiota, and ASD severity, we first identified several known butyrate-producing or pathogenic genera and assessed for correlations between such indices via *MaAsLin2*. We found that plasma IFN-γ levels are positively correlated with *Pseudomonas*, *Streptomyces*, and *Clostridium* relative abundances and negatively correlated with *Blautia* relative abundance (FDR-adjusted *p* < 0.1, Figures 5A–D). Additionally, plasma concentration of IL-6 is positively correlated with relative abundance of *Bacillus* (FDR-adjusted *p* < 0.1, Figure 5E). Subsequent correlational analysis was performed between plasma cytokine levels and gut microbiota relative abundances in subjects with ASD and behavioral symptom severity as assessed by CARS total score via Spearman’s rank correlation. Among the assessed cytokines, IL-4 was found to be significantly negatively correlated with CARS total score (Spearman’s ρ = −0.34, *p* < 0.05). All such correlations are uniquely observed in the ASD group. While no significant correlations were identified between gut microbiota abundance and CARS total score in subjects with ASD, we found that the identified gut microbiome dysbiosis signatures of ASD in the present study can classify the severity of ASD. Treating the CARS scoring categories *severe* and *mild-to-moderate* as a binary response for the logistic regression model and ROC curve analysis. Model features were selected from identified dysbiotic taxa in the ASD gut microbiome, including *Bacteroides*, Lachnospiraceae, *Clostridium*, *Desulfovibrio*, *Streptococcus*, *Neisseria*, *Bacillus*, and *Streptomyces*. The fitted logistic regression model is summarized in Supplementary Table 1. Using the identified microbiota associated with gut microbiome dysbiosis, we found that the ROC curve resulted in an AUC of 0.804 (95%CI = 0.653–0.918, Supplementary Figure 3).

**FIGURE 6:**
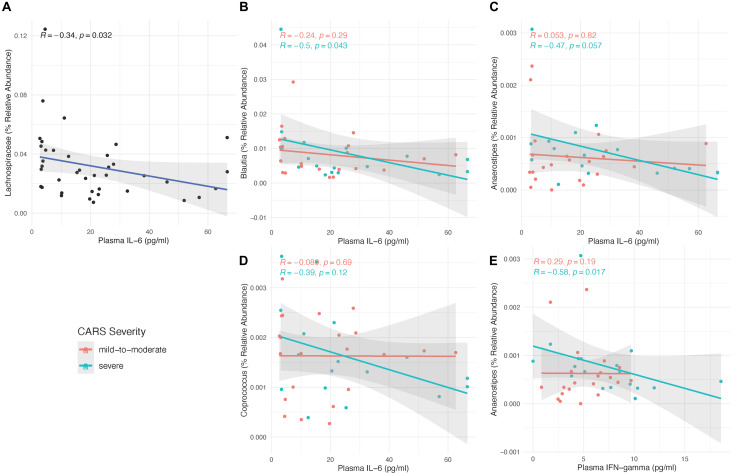
Relative abundance of Lachnospiraceae and associated genera are negatively correlated with plasma pro-inflammatory cytokines IL-6 and IFN-γ in subjects with ASD via Spearman’s rank correlation. **(A)** Family level Lachnospiraceae relative abundance is negatively correlated with plasma levels of IL-6 in subjects with ASD (*n* = 39). **(B–E)** Genus level associations between IL-6, IFN-γ, and relative abundances of Lachnospiraceae genera are significantly correlated in subjects with severe ASD (*n* = 17), but not in those with mild-to-moderate ASD (*n* = 22), as defined by CARS.

With a focus on the interactions between pro-inflammatory cytokines and butyrate producing microbiota, we then examined the associations between Lachnospiraceae genera and plasma levels of IL-6, IFN-γ, TNF-α, and TNF-β. We found that Lachnospiraceae family level relative abundances are correlated with plasma levels of IL-6 in subjects with ASD (Figure 6A, Spearman’s ρ = −0.34, *p* < 0.05). ASD severity subgrouping analysis as defined by severity categories based on CARS suggests that *Blautia* is significantly negatively correlated with plasma IL-6 levels in subjects with severe ASD (Figure 6B, Spearman’s ρ = −0.34, *p* < 0.05) and is not observed in the subjects with mild-to-moderate ASD severity. Despite the lack of statistical significance, several other known butyrate-producing genera belonging to the Lachnospiraceae family, including *Anaerostipes* and *Coprococcus*, also suggested more pronounced negative correlations with IL-6 only among those with severe ASD (Figures 6C,D). A similar correlational analysis between IFN-γ and Lachnospiraceae genera suggested that *Anaerostipes* was also found to be significantly negatively correlated with plasma IFN-γ levels in subjects with severe ASD (Figure 6E, Spearman’s ρ = −0.58, *p* < 0.05).

## Discussion

As a greater body of evidence points toward elevated inflammatory states and immune dysfunction as key characteristics of ASD pathophysiology, we first investigated differences between the immune cytokine profiles of individuals with ASD and healthy controls. We found that subjects with ASD possessed significantly higher plasma levels of IL-2, IL-4, IL-5, IL-6, IL-10, TNF-α, TNF-β, and IFN-γ when compared to healthy controls, implicating aberrations in immune cytokine profile to the extent of inducing a cytokine storm. IL-6, TNF-α, TNF-β, and IFN-γ are well-known to be involved in pro-inflammatory signaling (Turner et al., 2014a). IFN-γ was reported to activate glial cells and inflammatory cascade in brain (Sochocka et al., 2017). Additionally, IL-6 and TNF-α have been shown to be permeable to the blood-brain barrier, with potential interactions with the hypothalamus to induce subjective feelings of sickness and associated behaviors as a self-defense strategy during fevers in response to an infection (Dantzer, 2001, 2009; Dantzer et al., 2008). Since IL-6 and TNF-α are recognized as molecular signals of sickness upon interactions with the brain, such mechanisms likely contribute to the cognitive and behavioral deficits observed in ASD. IL-2, IL-4, and IL-5 are known modulators of adaptive immunity (Turner et al., 2014a). In particular, overexpression of IL-4 has been previously found to be associated with the onset of allergies, which is an inflammatory condition found to be more common in ASD (Oliphant et al., 2011; Chen et al., 2013; Masi et al., 2017). We found that IL-4 is significantly negatively associated with ASD severity via CARS total score, which likely indicates that allergy as an ASD co-morbidity is more common in those with mild-to-moderate ASD rather than severe ASD. IL-10, an anti-inflammatory signaling molecule, was also significantly higher in subjects with ASD than control, which could be a compensatory response of overactivation of inflammatory cytokines (Turner et al., 2014b). The higher plasma levels of IL-4 and IL-10 observed in our subject population may further implicate clinical subtypes of immune aberrancies among individuals with ASD, as both observed trends contradict previous findings in studies involving ASD (Masi et al., 2015).

With several previous studies that have observed elevated plasma levels of IL-1β, IL-8, and IL-12 p70 in children with ASD relative to healthy controls, we did not observe any significant groupwise differences between such cytokine plasma concentrations in the present study (Goines and Ashwood, 2013). Previously, elevation of IL-1β has been regarded as an indicator of inflammation and has been shown to be associated with obesity-induced insulin resistance (Ballak et al., 2015). Such results are consistent with our finding that BMI is significantly elevated in individuals with ASD and is positively associated with plasma IL-1β levels in all subjects with borderline significance in the ASD group (Supplementary Figures 1, 2). Interestingly, we did not observe a statistically significant elevation in plasma IL-1β in ASD group subjects when compared to healthy controls. While our results indicate no significant differences in plasma IL-1β expression between individuals with ASD and healthy controls, previous studies have found elevated expression of intracellular IL-1β in T cell immunoglobulin and mucin-domain-containing molecule 3 (TIM-3)-producing PBMCs, which has been proposed to contribute to neuronal dysregulation (Ahmad et al., 2019a). Elevated levels of IL-1β expression within monocytes have also been linked to pro-inflammatory effects and oxidative/nitrative stress, which is proposed to be mediated by a leucine zipper transcription factor known as the nuclear factor erythroid 2-related factor 2 (Nrf2) (Nadeem et al., 2020a). Thus, it is likely that the observed non-significant difference in mean IL-1β concentration between individuals with ASD and healthy controls indicate immune-compromised subtypes of ASD due to the multifactorial mechanisms of aberrant inflammation induction.

As one of the main goals of this study, we identified several differentially abundant taxa in the gut microbiome of subjects with ASD as compared to healthy controls. Many known pathogenic genera present in the gut of individuals with ASD were significantly higher in abundance while healthy control group subjects either had absent or significantly lower levels of such pathogenic genera; the relevant genera include *Clostridium*, *Desulfovibrio*, *Streptococcus*, *Neisseria*, *Bacillus*, *Proteus*, and *Streptomyces*. Among the identified pathogenic taxa, several species of the *Proteus* and *Neisseria* genera are known human pathogens that can induce diseases such as meningitis and urinary tract infections. *Streptomyces* and *Desulfovibrio* have been well documented in inducing inflammatory diseases and have been shown to produce toxic, pro-inflammatory compounds as secondary metabolites (Finegold et al., 2010; Herbrík et al., 2020). Containing more than 500 species, the colonization of *Streptomyces* within the human gut has been scarcely reported and evidence of its detection within that of ASD individuals has not been previously described. Nonetheless, species of the *Streptomyces* genus is known for inducing invasive infections in those who are immunocompromised and its spores have shown to provoke lung inflammation and systemic immunotoxicity (Jussila et al., 2003). Therefore, we believe that the presence of detected *Streptomyces* may provide additional evidence in the elucidation of dysbiotic events in the ASD gut flora and implicates additional mechanistic causes of pro-inflammatory states within the ASD gut. Additionally, several species of the *Clostridium* genus, such as *C. histolyticum* group (*Clostridium* clusters I and II) of bacteria, have previously been found to be elevated in the stool of subjects with ASD relative to healthy controls, which is consistent with results of the present study (Parracho et al., 2005). Past studies have suggested that such *Clostridia* species are known to secrete neurotoxin molecules that can enter systemic circulation and elicit harmful effects on remote organs and tissues, such as the brain, while additionally being associated with more localized GI problems in subjects with ASD when present in high abundances (Parracho et al., 2005; Argou-Cardozo and Zeidán-Chuliá, 2018).

We found that gut microbiome of subjects with ASD harbor lower levels of Lachnospiraceae, which was also determined to be negatively correlated with plasma IL-6 levels in subjects with ASD. Further analysis at the genus level suggests that the *Blautia* genus is significantly negatively correlated with plasma IL-6 levels in subjects with more severe symptoms of ASD. Despite the lack of statistical significance, several other known butyrate-producing genera belonging to the Lachnospiraceae family, such as *Coprococcus* and *Anaerostipes*, also suggested more pronounced negative correlations with IL-6 among those with severe ASD symptoms. Interestingly, the gut microbiome correlation network of healthy controls was observed to harbor several closely associated butyrate-producing Lachnospiraceae genera when ranked by the node’s degree (i.e., number of connected edges), such as *Roseburia*, *Coprococcus*, *Blautia*, and *Anaerostipes.* Despite being central to the microbiome in healthy subjects, such Lachnospiraceae genera are not found among the more dominant nodes in the microbial network for subjects with ASD. Thus, we attribute the dramatic increase in associations among overall nodes observed in the ASD microbiome to the proliferation of largely pathogenic microbiota and gut microbiome dysbiosis. As gut microbiome dysbiosis is known to be associated with the pathogenesis of GI disorders, we hypothesize that restoration of healthy levels of Lachnospiraceae within the ASD gut microbiome may help alleviate gut inflammation and associated GI symptoms (Carding et al., 2015; Fattorusso et al., 2019). Furthermore, a previous study by Zhang et al. (2019) have suggested that species of the Lachnospiraceae family show beneficial effects in rats with stress-induced visceral hypersensitivity. Visceral hypersensitivity is a known cause of functional GI disorders and is associated with GI disturbances commonly observed in ASD (Delgado-Aros and Camilleri, 2005; Wasilewska and Klukowski, 2015). Taken together, past findings and results from the current study suggest that altered levels of bacteria belonging to the Lachnospiraceae family are defining features of ASD gut microbiota dysbiosis and contribute to the high prevalence of functional GI disorders found in individuals with ASD. Furthermore, our results suggest that dysbiotic gut microbiota can serve as predictors of ASD severity, as shown by the performance of classification of CARS severity in individuals with ASD based on all disturbed pathogenic and beneficial bacteria (Supplementary Table 1 and Figure 3, AUC = 0.804, 95%CI = 0.653–0.918).

With several pro- and anti-inflammatory signaling cytokines and adaptive immunity modulators found to be elevated in plasma levels among subjects with ASD compared to healthy controls, our results suggest a significant immune disturbance and inflammation in subjects with ASD. With a large body of evidence suggesting the anti-inflammatory effects of butyrate, we identified specific gut microbiome taxa associated with butyrate production and their correlations with plasma levels of pro-inflammatory cytokines. The Lachnospiraceae family of microbiota was found to exist in reduced abundances within the gut of individuals with ASD, which has also been found in past studies involving individuals with ASD (Ma et al., 2019; Vacca et al., 2020). Lachnospiraceae constitutes a family of known SCFA-producing species with *Blautia* and *Roseburia* being the major SCFA-producing genera within the family, representing one of the main regulators of gut inflammation (Vacca et al., 2020). Given that the gut microbiome plays a significant role in the regulation of host immune function and GI health, the metabolite secretions from such bacterial taxa thus represent a major mechanism by which the gut microbiota interacts with its host. With butyrate being the preferred substrate for absorption via colonocytes, SCFAs produced by gut microbiota have been shown to influence host neuroimmunoendocrine function by uptake in the colon where it subsequently participates in systemic circulation with a potential to cross the blood-brain barrier and directly modulate brain function (Topping and Clifton, 2001; Treuren and Dodd, 2017; Silva et al., 2020).

As previous studies have suggested the beneficial effects of butyrate on gut-associated immune function, we then investigated the correlations between butyrate-producing genera and pro-inflammatory cytokine levels within ASD group subjects (Corrêa-Oliveira et al., 2016). Based on our findings, the negative correlation between *Blautia* relative abundance and plasma IFN-γ levels in subjects with ASD thus suggests that a reduction in the major butyrate-producing genus is associated with an increase in the pro-inflammatory cytokine IFN-γ. Previously, butyrate has been shown to possess multiple mechanisms of action in the reduction of local inflammation in the intestinal mucosa. One of such mechanisms has suggested that the signaling pathways of highly upregulated IFN-γ levels in the IBD mucosa can be inhibited by the SCFA butyrate (Klampfer et al., 2003). Due to the high prevalence of comorbid IBD in subjects with ASD, the reduction in butyrate-producing species may provide insight into factors that contribute to local inflammatory processes commonly associated with ASD. Furthermore, the observed negative correlation between *Blautia* relative abundance and the concentration of IFN-γ in peripheral blood is consistent with our hypothesis that known etiopathogenic mechanisms of ASD are, at least in part, associated with the reduction of gut butyrate-producing species’ abundance.

To further elucidate the association between altered abundances of butyrate-producing species within the ASD gut microbiome and inflammation, we characterized predicted biological pathways mediated by the gut microbiome and explored the association between systemic inflammatory cytokine levels with the gut flora. Based on our results, we first determined that microbial dysbiosis in subjects with ASD may lead to aberrant levels of microbial fatty acid metabolism. The overall fatty acid biosynthesis by gut microbiota in subjects with ASD is significantly reduced relative to that of healthy subjects while the abundance of bacteria known to produce unsaturated fatty acids is elevated. Thus, we infer that the microbiota within the gut of subjects with ASD contains dramatically reduced proportions of microbiota known to produce saturated fatty acids while such proportions are significantly higher in healthy subjects. Such results suggest that bacteria known to produce SCFAs (particularly butyrate) are significantly reduced in the gut of subjects with ASD, which is consistent with parallel results determined based on the summed relative abundances of known butyrate-producing taxa. Additionally, previous studies have found that elevated levels of other non-SCFA anionic species, such as lactate, when present in feces have led to a subsequent decrease in levels of fecal SCFAs (Mortensen and Clausen, 1996). Such a phenomenon may suggest that elevated abundances of lactate-producing bacteria within the ASD gut microbiome, such as the *Lactobacillus* genus, may additionally reduce SCFA production in the gut of individuals with ASD, which is consistent with our findings (data not shown). It is well documented that butyrate can ameliorate intestinal mucosal inflammation, reinforce the epithelial defense barrier, and modulate visceral sensitivity and intestinal motility (Canani et al., 2011). Thus, a deficit in such beneficial butyrate-producing bacteria may represent a mechanism of induction of inflammatory diseases within the ASD population.

## Conclusion

Based on the findings of the present study, the observed associations between dysbiotic gut microbiota composition and aberrant immune cytokine profiles in individuals with ASD suggests a potential mechanistic linkage between dysbiotic levels of specific butyrate-producing gut microbiota and the overexpression of immune cytokines. We propose that the observed reduction in the amounts of gut butyrate-producing genera may possess a mechanistic role in the induction of inflammatory diseases within the ASD population. Therefore, it remains hopeful that therapeutic approaches attempting to restore a healthy gut microbiome composition, such as through the administration of probiotics or fecal transplants, may help to improve clinical symptoms of ASD. However, the results of the current study are limited as the quality of 16S sequencing data for control group stool samples requires further validation. Thus, microbial abundances corresponding to the subjects within the healthy control group should be interpreted with caution. Secondly, the study was conducted within a homogeneous cohort of Chinese participants. As such, conclusions drawn based upon such results should be further validated within a racially unbiased subject population. Lastly, comparison of age and BMI between groups suggested a significant groupwise differences between the ASD group and controls. We attribute the difference in age between groups to the enrollment of four teenaged subjects within the ASD group (8.89% of total ASD group subjects), three of which also correspond to outliers based on BMI (Supplementary Figure 1). Future studies are needed to further validate the generalizability of findings within the present study in terms of age, race, and weight-associated characteristics such as BMI. We hope that the present study serves as inspiration for future research on the potential to use butyrate-producing probiotic species to alleviate both localized and systemic inflammation involved in several ASD-associated comorbidities.

## Data Availability Statement

The datasets presented in this study can be found in online repositories. The names of the repository/repositories and accession number(s) can be found in the article/Supplementary Material.

## Ethics Statement

This study was approved by the Institutional Review Board (IRB) of The Second Affiliated Hospital of Kunming Medical University (Review-YJ-2016-06). Written informed consent to participate in this study was provided by the participants’ legal guardian/next of kin.

## Author Contributions

X-JK and ZN performed conceptualization. KL, HW, and LX performed data curation. KL and XC performed formal analysis. XC, PW, YZ, ZL, JW, CS, and ML performed investigation. X-JK, XC, and KL performed methodology. XC and X-JK performed funding acquisition, project administration, and performed resources. ZN, JL, XC, and X-JK performed supervision. KL performed software, validation, and visualization. KL and ZN performed writing – initial draft; X-JK, KL, JL, and Y-WL performed writing – review and editing. All authors contributed to the article and approved the submitted version.

## Conflict of Interest

The authors declare that the research was conducted in the absence of any commercial or financial relationships that could be construed as a potential conflict of interest.
