# Supplementary figures and images for: Dysbiotic Gut Microbiota and Dysregulation of Cytokine Profile in Children and Teens With Autism Spectrum Disorder
Source: Front Neurosci. 2021 Feb 10;15:635925. doi: 10.3389/fnins.2021.635925 (PMC7902875; doi:10.3389/fnins.2021.635925)

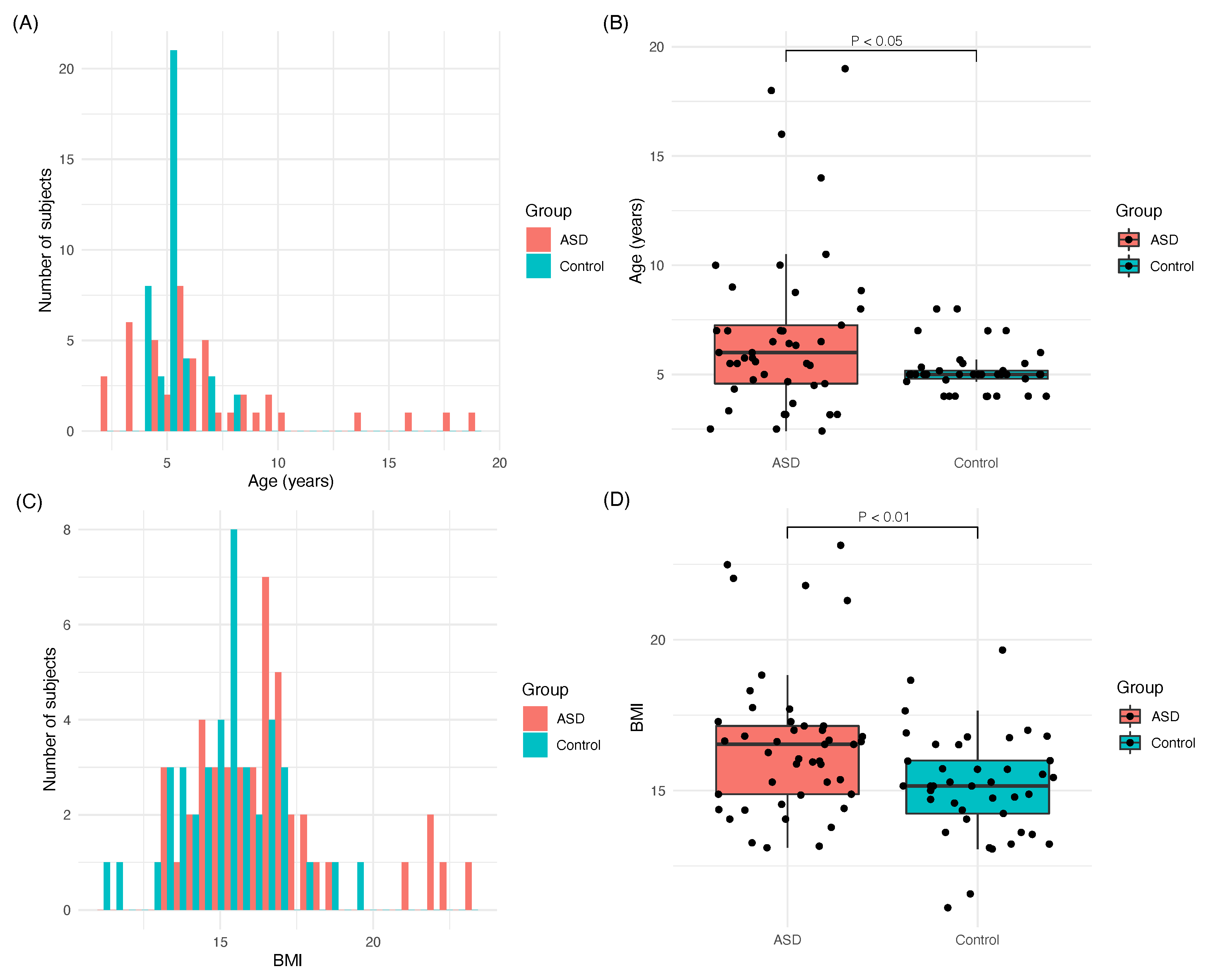

Supplement: Supplementary file 1 [file Image_1.TIFF]

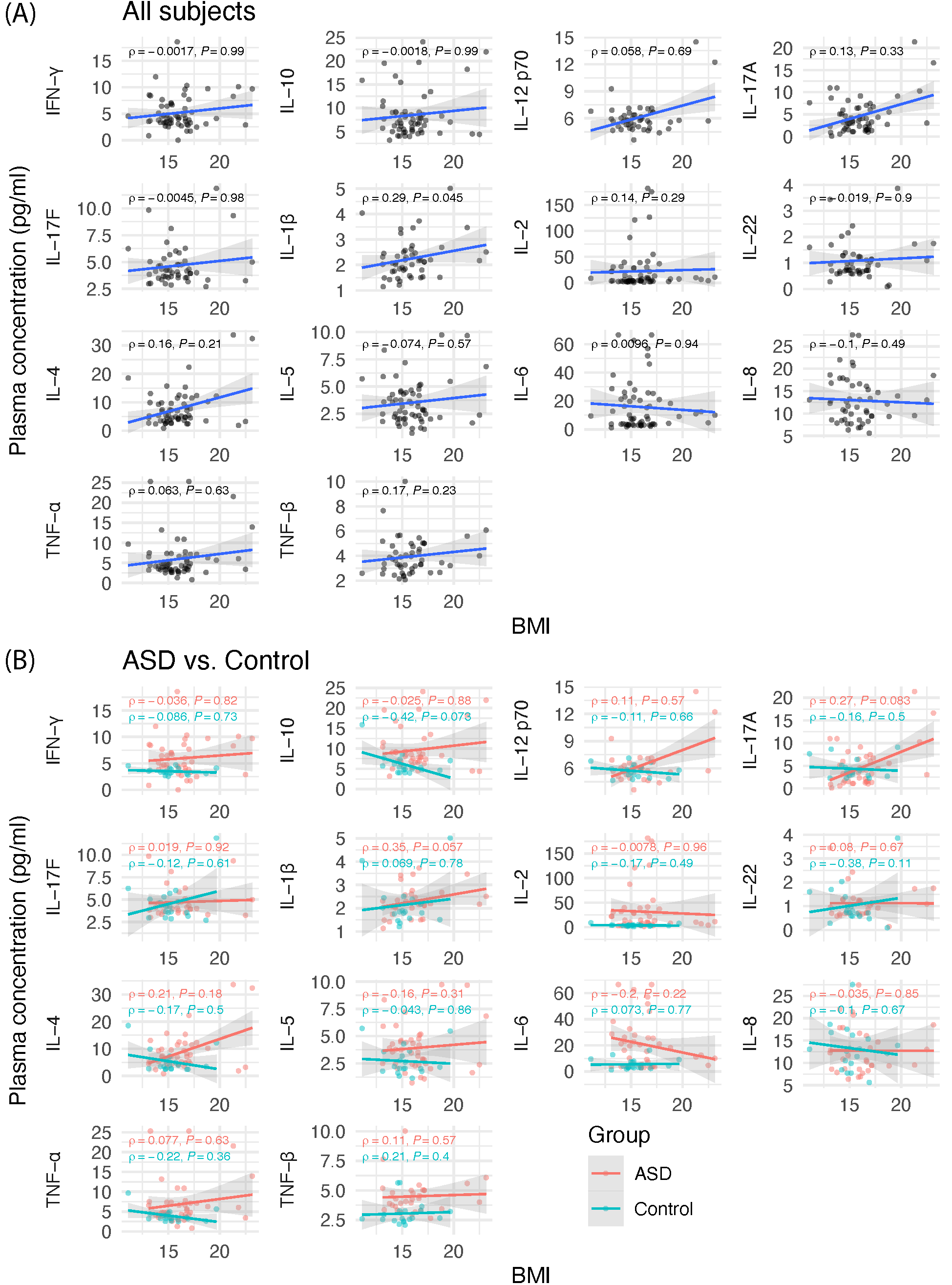

Supplement: Supplementary file 2 [file Image_2.TIFF]

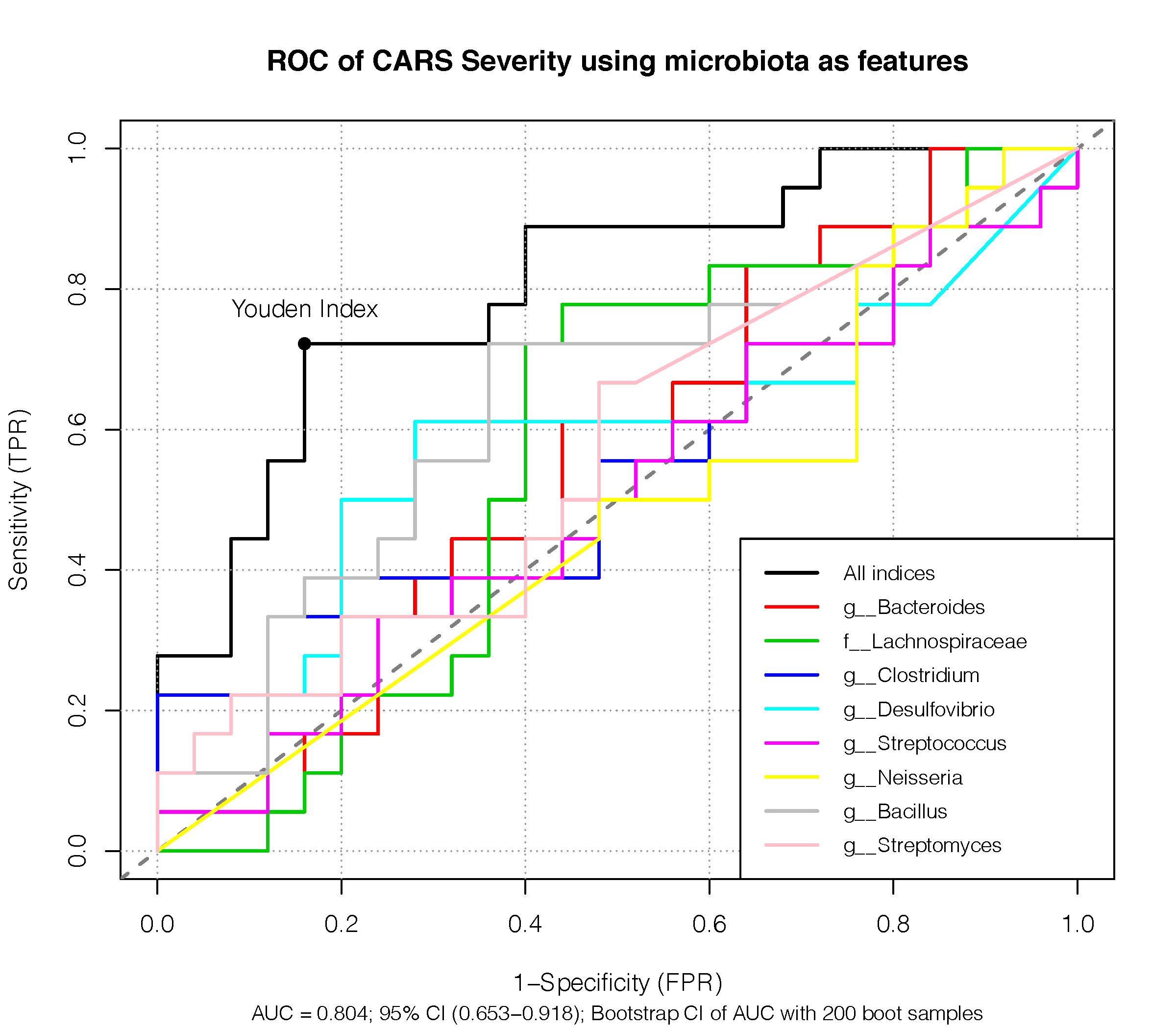

Supplement: Supplementary file 3 [file Image_3.TIFF]
