# Supplementary material for: Dysbiotic Gut Microbiota and Dysregulation of Cytokine Profile in Children and Teens With Autism Spectrum Disorder
Source: Front Neurosci. 2021 Feb 10;15:635925. doi: 10.3389/fnins.2021.635925 (PMC7902875; doi:10.3389/fnins.2021.635925)
Supplement: Supplementary file 4 [file Table_1.DOCX]

Supplementary Material

# Supplementary Figures and Tables

## Supplementary Figures


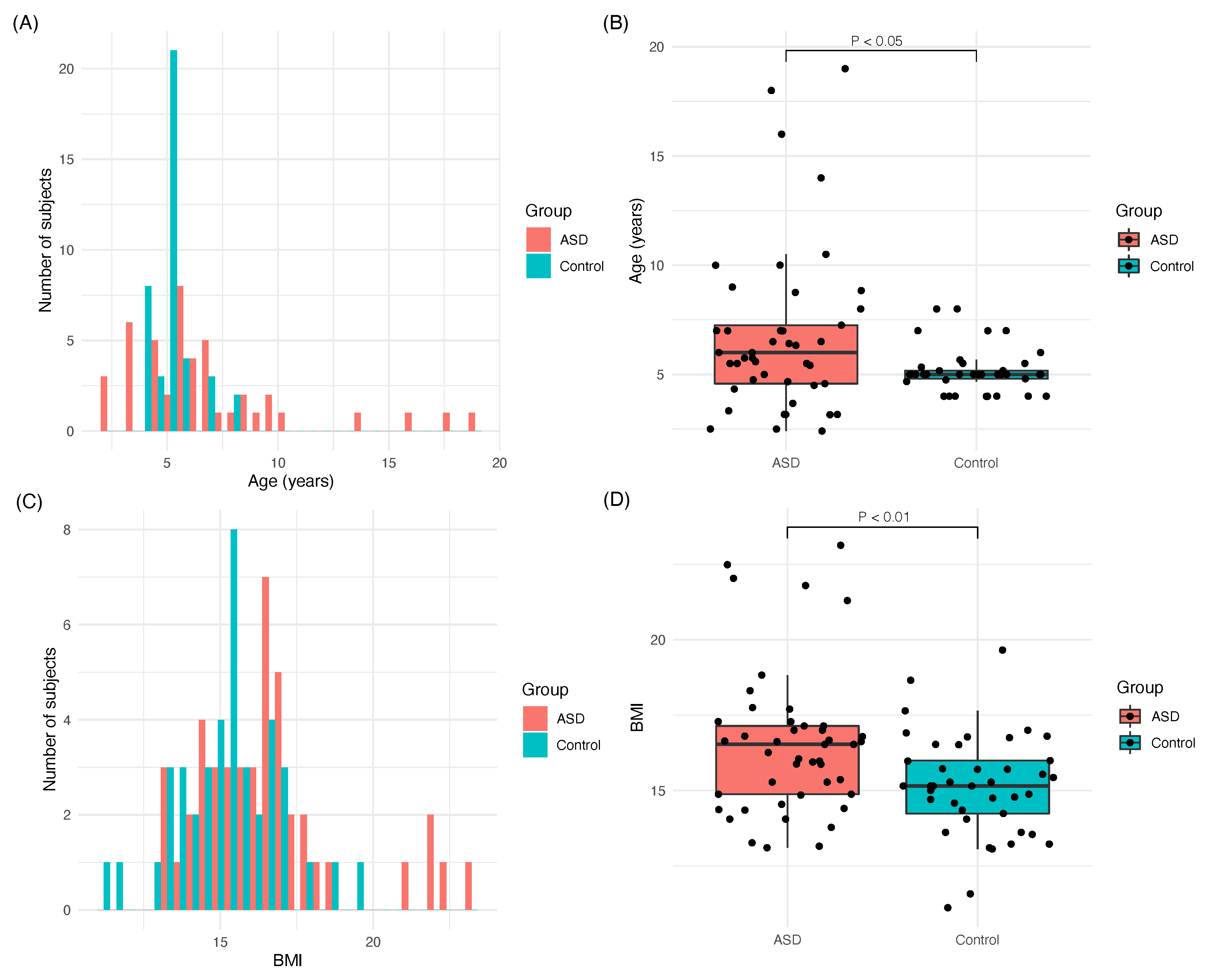


**Supplementary Figure 1.** Overview of study participant age and body mass index (BMI) distributions and groupwise comparisons in individuals with ASD (*n* = 45) and healthy controls (*n* = 41). (A) Age distribution in all subjects, colored by group. (B) Mean age is significantly higher in individuals with ASD when compared to healthy controls via Wilcoxon rank-sum test (*P* < 0.05). (C) BMI distribution in all subjects, colored by group. (D) Mean BMI is significantly higher in individuals with ASD when compared to healthy controls via Wilcoxon rank-sum test (*P* < 0.01).


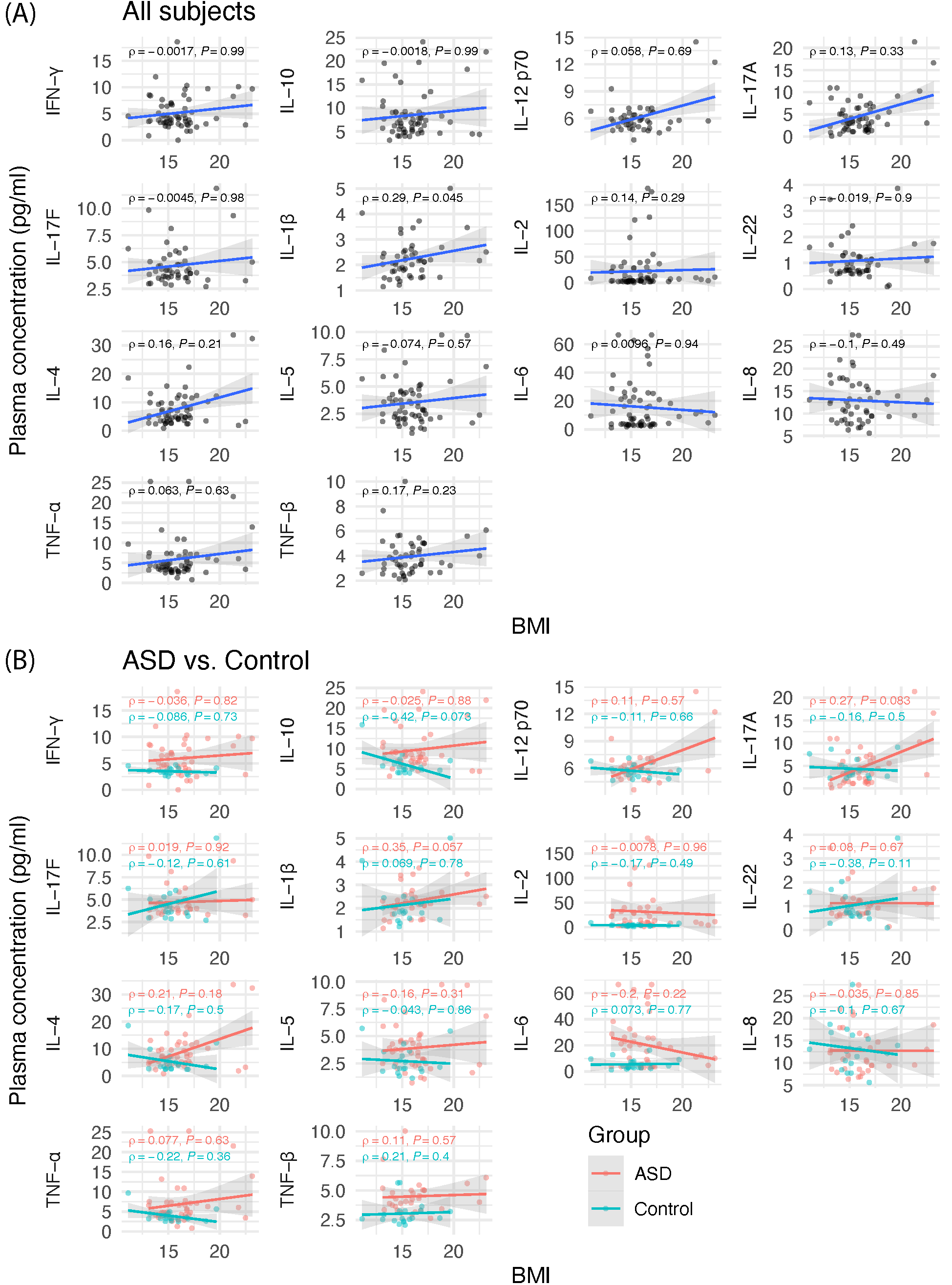


**Supplementary Figure 2.** Associations between plasma cytokine concentrations and body mass index (BMI). (A) Spearman’s rank correlation shows that BMI is positively associated with plasma IL-1β levels in all subjects (*P* < 0.05). (B) Groupwise assessment of correlations reveals that the correlation between BMI and IL-1β are not statistically significant in healthy controls with borderline significance in ASD group (*P =* 0.057).


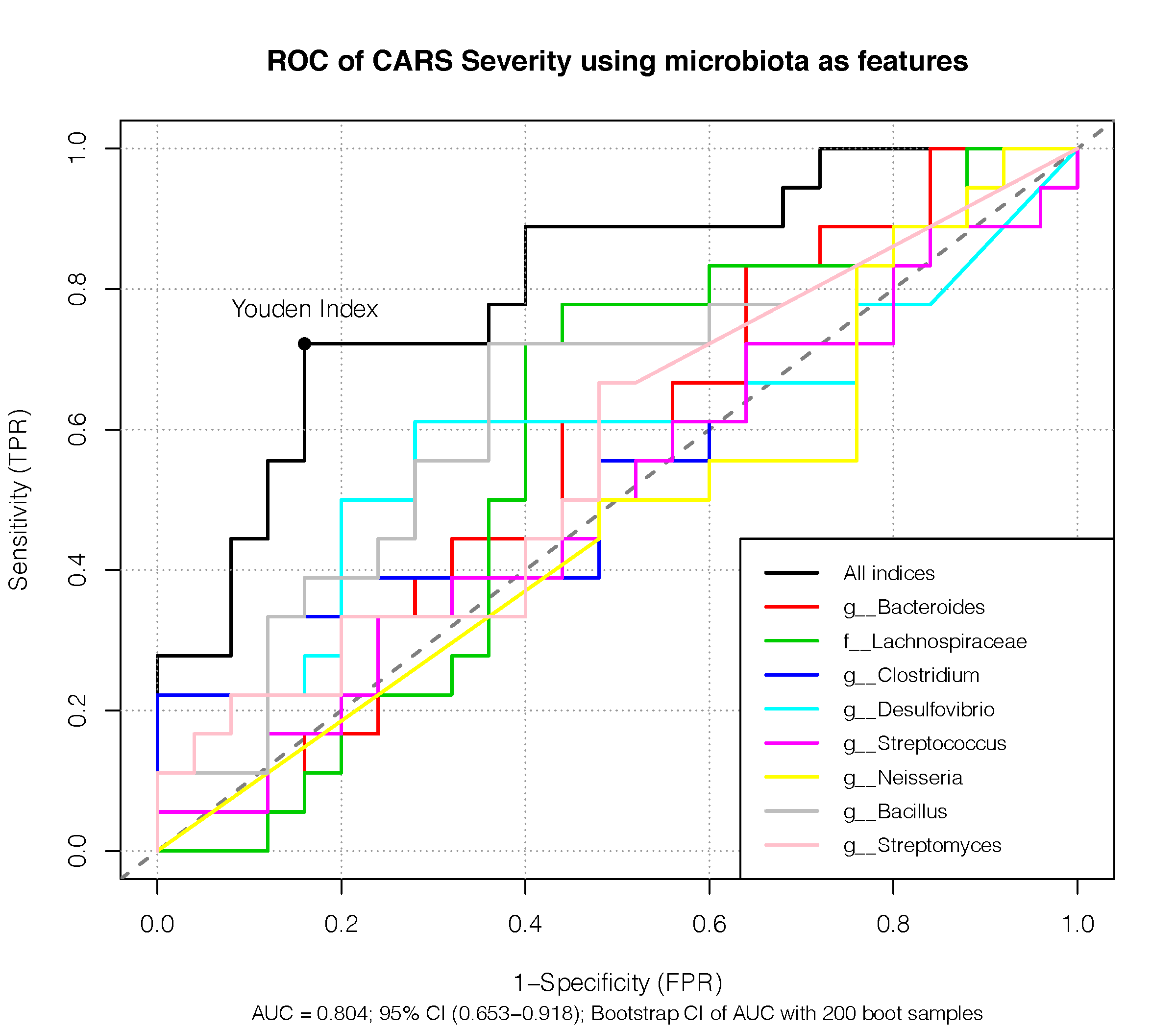


**Supplementary Figure 3.** Logistic regression reveals that identified dysbiotic microbiota can classify CARS ASD severity. Using all identified dysbiotic microbiota relative abundance to predict ASD severity based on CARS severity categories (*mild-to-moderate* and *severe*, treating *severe* as a case and *mild-to-moderate* as control) resulted in an AUC of 0.804 (95% CI: 0.653-0.918). Confidence interval of the AUC is determined based on 200 bootstrapping iterations.

## Supplementary Tables

**Supplementary Table 1.** Summary of CARS severity logistic regression model indices used in ROC analysis.

| **Taxa** | **Estimate** | **Standard Error** | ***z*-value** | ***P*-value** |
| --- | --- | --- | --- | --- |
| *Bacteroides* | 5.278 | 3.131 | 1.686 | 0.0919 |
| Lachnospiraceae | -8.088 | 20.733 | -0.390 | 0.6965 |
| *Clostridium* | 5.072 | 470.856 | 0.011 | 0.9914 |
| *Desulfovibrio* | 366.888 | 382.338 | 0.960 | 0.3373 |
| *Streptococcus* | 122.921 | 81.473 | 1.509 | 0.1314 |
| *Neisseria* | -13257.180 | 7940.707 | -1.670 | 0.0950 |
| *Bacillus* | 3656.716 | 1884.956 | 1.940 | 0.0524 |
| *Streptomyces* | 1697.280 | 1860.408 | 0.912 | 0.3616 |
